# Skewed Exposure to Environmental Antigens Complements Hygiene Hypothesis in Explaining the Rise of Allergy

**DOI:** 10.1007/s10441-017-9306-7

**Published:** 2017-03-24

**Authors:** Wilfried Allaerts, Tse Wen Chang

**Affiliations:** 1 Biological Publishing A&O and Immunology Department, Erasmus MC, Rotterdam, CA The Netherlands; 2 Genomics Research Center, Academia Sinica, Taipei, 11529 Taiwan

**Keywords:** Asthma, Allergic diseases, Skewed antigen exposure, Hygiene Hypothesis, Domestic environment biodiversity

## Abstract

The Hygiene Hypothesis has been recognized as an important cornerstone to explain the sudden increase in the prevalence of asthma and allergic diseases in modernized culture. The recent epidemic of allergic diseases is in contrast with the gradual implementation of *Homo sapiens sapiens* to the present-day forms of civilization. This civilization forms a gradual process with cumulative effects on the human immune system, which co-developed with parasitic and commensal Helminths. The clinical manifestation of this epidemic, however, became only visible in the second half of the twentieth century. In order to explain these clinical effects in terms of the underlying IgE-mediated reactions to innocuous environmental antigens, the low biodiversity of antigens in the domestic environment plays a pivotal role. The skewing of antigen exposure as a cumulative effect of reducing biodiversity in the immediate human environment as well as in changing food habits, provides a sufficient and parsimonious explanation for the rise in allergic diseases in a highly developed and helminth-free modernized culture. Socio-economic tendencies that incline towards a further reduction of environmental biodiversity may provide serious concern for future health. This article explains that the “Hygiene Hypothesis”, the “Old Friends Hypothesis”, and the “Skewed Antigen Exposure Hypothesis” are required to more fully explain the rise of allergy in modern societies.

## Introduction

The postmodern rise in allergic diseases often has been imputed to the notion of increased hygiene standards and hence decreased microbial infections, the so-called ‘Hygiene Hypothesis’(Strachan 1989). Allergic diseases are now affecting 20–40% of the population of highly industrialized, economically advanced regions of the world. The incidence of these disorders appears to have dramatically increased in the United States of America and Europe, but also in developed Asian countries (Rook and Brunet 2005; Chang et al. 2007; Versini et al. 2015). Changes of lifestyle are most evident among young children, and therefore, children are the most vulnerable to develop allergic diseases (Versini et al. 2015). Very recently, a detailed account of the historical recordings of the allergy epidemics, from the first reported hay fever case in 1870 until 2010, was published by Platts-Mills (2015).

In order to understand the adaptability of human nature towards a modernized culture, one has to consider the human adaptation in an evolutionary perspective. The history of highly industrialized, postmodern civilization, however, has but a span of at most one century, and therefore, represents merely a few millionth parts of 1% of the evolution of mammals. In evolutionary perspective, one could also say this civilization is a brand new change of lifestyle but not-yet-adapted to our natural environmental conditions. Allergic reactions do not belong to the well-adapted functions of the immune system, because they are harmful to animals. Since the early branching of vertebrate taxa during evolution, some 300 million years ago, vertebrates have been living in close association with a variety of Helminths (non-systematic group name of parasitic worms of the phyla *Annelida*, *Platyhelminthes*, *Nematoda* and *Acanthocephala*). In fact, their cohabitation with helminthic worms had some advantage for their immune system. Therefore, it may be described as a form of commensalism, a relationship between two species in which one obtains nutritional substances or other benefits from the other without damaging or exhausting the other. This commensalism was also helpful to their evolutionary success. This was elegantly described by Graham A.W. Rook as the ‘Old Friends Hypothesis’ (Rook and Brunet 2005; Rook 2013).

The first, hard evidence of a link between modern hygienic conditions and the prevalence of allergy was demonstrated by Strachan (1989), showing an inverse correlation between the numbers of older siblings in the household and the prevalence of hay fever. However, Strachan (1989) did not make an explicit connection to the changes in the biodiversity of the domestic environment. The dramatic reduction in the biodiversity of house, food, soil and skin micro-organisms was not the focus of his article (Strachan 1989), but also not in the recent review by Platts-Mills (2015). Nonetheless, a rational explanation of the increased prevalence of IgE-mediated diseases and their clinical manifestation cannot be reduced to modern behavioral changes *in se.* In particular, it needs a careful examination of the involvement and dynamics of antigen-specific IgE-cross-linking and postreceptor signal transduction (Chang et al. 2007; Chang 2000). On the other hand, the first connection between a low prevalence of autoimmune diseases and hygiene status in an underdeveloped population of Nigeria was already suggested by Greenwood (1968). Strachan’s thesis, stating that the dramatic reduction of infections due to a ‘better’ hygiene was countered by an impaired balance and increase in immune-mediated diseases, was demonstrated by a large scale cohort study of more than 17,000 children born in 1958, followed for 23 years (Versini et al. 2015).

In modern societies, the dramatic rise of allergy prevalence is directed towards a limited number of specific, non-infectious environmental substances. For most patients residing in a local region, they are sensitive to less than a few dozens of antigens among the millions of environmental substances, and among those, dust mites, animal dander, pollens of a few species of trees, grasses and weeds are prominent. This phenomenon was explained by Tse Wen Chang (Chang and Pan 2008; Chang 2014) to be caused by a changing pattern of antigen exposure, called the ‘skewed antigen exposure’ hypothesis, in addition to the Hygiene Hypothesis. In this paper, we will investigate the arguments for this ‘skewed antigen hypothesis’ and demonstrate that this hypothesis may fulfill the criterion of parsimony to explain the increased prevalence of allergic diseases in a helminths-free, postmodern culture. Moreover, we will extend our analysis to include the effects of increased hygiene, behavioral effects and changes in postmodern food consumption on the immune responsiveness of modern beings. Finally, well-established therapies for the cure and prophylaxis of allergic diseases will be suggested and discussed.

## Role of IgE in the Human Immune System and During Evolution (*Compared to IgA*)

Immunoglobulin E (IgE) constitutes a class of antibodies of the adaptive immune system, discovered simultaneously by the groups of Ishizaka et al. (1967) and Johannson and Bennich (1967). Normally it is present in the serum at very low concentrations (IgE is in the range of 100–500 ng/ml, so compared to the range of IgG, being 10–15 mg/ml, the concentration of IgE is much lower) (Abbas and Lichtman 2005). It is produced by IgE-secreting plasma cells (terminally differentiated B-cells), which express the antibody on their surface during their maturation (Chang 2000). The increased amount of IgE-production in humans infected with helminthic worms is still not well understood. These high levels of total serum IgE (~5000 IU/ml) were for instance found to be common among children living in rural areas in Venezuela (Lynch et al. 1998) and Indonesia (Hamid et al. 2013), whereas the total serum IgE in children in modern societies is around 100 U/ml (only 2.0% of the former) (Wittig et al. 1980). However, the increased ratio of allergen-specific IgE to total IgE in allergic patients is a strange observation, which so far is not fully comprehended (Chang 2014; Lynch et al. 1998). Allergic patients thus produce increased amounts of IgE directed towards non-pathogenic, innocuous antigens to which they are sensitive.

Normally, IgE molecules circulate in the blood and bind to IgE-specific receptors on basophilic leukocytes in the circulation and mast cells along the mucosal linings and underneath the skin (Chang 2000). In allergic reactions, the inhaled or ingested allergens bind to IgE molecules (at least several hundreds) on mast cells or basophils, which results in cross-linking of the IgE-molecules. This cross-linking of IgE-molecules triggers the aggregation of underlying high-affinity receptors (FcεRI) on mast cells and basophils (Chang 2000), resulting in the release of biogenic amines (e.g. histamine) and lipid mediators (e.g. leukotrienes) and, as a result, an immediate hypersensitivity reaction.

It is important to note some differences with IgA-mediated immune reactions here. Unlike IgE, the IgA-molecules have no receptors on mast cells or basophils. In the blood, IgA molecules, also secreted by plasma cells, are bound by FcαR (CD89) on neutrophils, eosinophils and monocytes (Abbas and Lichtman 2005). Binding of IgA by eosinophils may result in IgA-dependent cell-mediated cytotoxicity (Abbas and Lichtman 2005). IgA molecules do however consist in different forms (isotypes and oligomers) (Delacroix et al. 1982; Simell et al. 2006). Oligomeric IgA’s (a polypeptide of around five times the IgA mass) are found in external mucosal secretions. They are taken up by endocytosis at the basolateral surface of epithelial cells. After proteolysis from their receptor, the undegraded IgA can be transported across the cells into secretions (like tears, saliva, gut fluid) (Kaetzel et al. 1991). In the colostrum (mother milk) also both isotypes of IgA are found (IgA1 and IgA2). IgA’s can also bind to the mucus layer on top of the epithelial cells and form a protective barrier against antigenic moieties present in the gut lumen.

Although most cases of allergic reaction are not life-threatening, in some cases they may however result in acute conditions, like a life-threatening anaphylactic shock. Allergic hypersensitivity has to be regarded as a dysfunctional adaptation, because it is not adding to the evolutionary fitness of animal species. They do not serve a defense or sentinel function, or even put the allergic animal at risk for predators, because it can make them for instance noisy when they wheeze and sneeze. Actually, allergic reactions are not known in wild animals, but they do occur in certain domesticated forms of mammal species that have similarly adapted to modern human lifestyle, like cats and dogs (Greenwood 1968; Chesney 2001; Verlinden et al. 2006). These are typically animals that also live within the closed, domestic environment, but do not necessarily adopt postmodern human behavior like computer gaming or watching television (Platts-Mills 2015) (so, these activities do not constitute a cause for allergic reactions by themselves). On the contrary, allergic diseases are absent in Chimpanzees (genus: *Pan*), which are human’s closest evolutionary relatives (Hobolth et al. 2007).

Whereas species within all mammalian groups (at taxonomic level of sub- or infraclasses) appear to possess a developed IgE immunoglobulin repertory and high-affinity receptor system (FcεRI) (Vernersson et al. 2004), both IgE and FcεRI are absent in birds, although birds do express most other immunoglobulin class molecules (IgA, IgG and IgM) and distinctive components of the vertebrate adaptive immune system (like MHC molecules, T-cell receptors, cytokines, etc.) (Chang and Pan 2008). Therefore, it is conceived that the evolutionary history of IgE-class immunoresponsiveness goes back at least 150 million years before present (mybp) (or the maximum estimated superordinal divergence times within mammals) (Waddell et al. 1999) to at most some 300–350 mybp, the divergence time when birds and mammals diverted from jawed vertebrate ancestors (Waddell et al. 1999; Benton 1990). Moreover, all vertebrate classes appear to use the primary elements of adaptive immunity, namely VDJ genes, recombinase activation genes, immunoglobulins, B and T cell receptors and MHC molecules (Cannon et al. 2004; Laird et al. 2000). IgE-mediated immune reactivity therefore, would be consistent with the view that it evolved particularly in mammalian taxa that lived as commensal species together with their helminthic visitors.

Finally, a genomic linkage between certain genes (like coding for the *Disintegrin and Metalloproteinase domain containing protein 33*, ADAM 33) in certain families with high prevalence of asthma have been suggested to implicate a genetically inherited mechanism (Cheng et al. 2004). However, studies on identical twins, often living in the same household and sometimes showing discordance with respect to their allergic status, corroborate the view that the environmental causes are dominant; genetic predisposition eventually could exacerbate the ill-adapted allergic reactions to these external agents.

## The Environmental Changes Since the Arrival of *Homo sapiens sapiens*

For a comprehensive study of human adaptability to its environment in an evolutionary perspective, a rather unfamiliar recourse is needed to miscellaneous evidence from palaeobiological (before culture), archaeological (unwritten culture) and scarcely available historical materials.

### The Early Hominoids and Origin of Human Civilization

#### Control of Fire and Shaping of the Immune System

Apparently, the modernization of humans started with the control of fire. The control of fire, however, was only the beginning of the human control of his surrounding environment. Doing so, man was able to cook his food and kill a lot of microbial organisms before ingestion. Consequently, the use of cooked food altered the contact of humans with their microbial environment and contributed to the shaping of their immune system.

Whereas some studies have revealed pieces of evidence suggesting that the first control of fire was achieved by the ancestral hominid *Homo erectus* about 790,000 years ago (Goren-Inbar et al. 2004), there is common agreement that widespread use of fire was implemented by *H. sapiens* around 125,000 years ago (James 1989). There is also ample evidence suggesting that the use of fire was adopted from the Neanderthal subspecies *Homo sapiens neanderthalensis*, which was particularly adapted to the colder climates of the Eurasian continent (Roebroeks and Villa 2011). The cultural transfer of fire control from *H.s. neanderthalensis* to *H.s. sapiens* (i.e. modern humans), touches upon the question of whether *H.s.s.* led to extinction of the Neanderthals some 40,000 years ago, or whether both subspecies had significantly interbred while co-habiting in a number of regions of the Eurasian continent (Fu et al. 2015). Interestingly, recent studies provided evidence for a genetic transfer of HLA-class I alleles from archaic humans (consisting of two sister groups, the Neanderthals and Denisovans) to modern humans in Europe, (East-)Asia and Oceania (Abi-Rached et al. 2011). These alleles, some of which encoding for unique or strong ligands for natural killer (NK) cells, appeared to be missing in the original African gene pool, but may have been introduced into Africans at a later stage (Roebroeks and Villa 2011). These data would be suggestive for the interbreeding hypothesis (Roebroeks and Villa 2011; Fu et al. 2015; Abi-Rached et al. 2011), and suggests that beside the use of fire also changes in immunological defense mechanisms took place when *H. sapiens* started to inhabit the colder regions of the northern hemisphere. It is still not known what happened among humans between 125,000 and 40,000 years ago.

#### The Invention of Clothing, Foot Wear and First Human Steps to Modernity

Much later, also other ‘inventions’ occurred that reduced the contact with infectious organisms. Examples hereof are the use of shoes (already around 40,000 years ago, as observed from a change in anatomy of the proximal pedal phalanges) (Trinkaus 2005) and man-made clothing (Fisher 1981). Even today, some regions of the pristine Amazon forest are inhabited by local tribes that do not wear shoes or extensive clothing. It is well known however, that many of these indigenous tribes became almost extinct due to the fightings or due to the contagious diseases that the contacts with Western discoverers and colonists procured them in the fifteenth and twentieth centuries (Olson 1991). With the use of fire, man could also fabricate other tools (iron-made in stead of stone-made), which led to numerous wars and conquests, and also led to a constant changing of the inhabited domains.

### Changes in the Domestic Environment

#### History of Hygiene and Sanitation

The changes in contacts between man and his biological and microbiological companions are most dramatically observed in the domestic environment. For instance, it is known that - much unlike our postmodern, industrialized societies - common houses in large parts of the so-called ‘civilized’ world didn’t have water-supplied sanitation until the Modern Ages (17th – 18th centuries). From historical sources (references not listed) it is well known that even in famous royal palaces like Versailles (Paris, France) modern sanitation was lacking. For the common people, however, these were unaccessible places. However, the Minoan civilization of Crete (around 1800 BC, at Knossos) was the first to utilize an underground system of clay pipes and a complex network of masonry for sanitation and water supply (for pressurized showers) (Anonymous 1989). Ancient cultures in the Indus Valley, Egypt and Persia were also using a water management system based on aquaducts (Persia) and copper pipes (Indus, Egypt), the latter used for privileged settlements only (Anonymous http://enlightenme.com/ancient_plumbing). The Persian ‘qanats’ (aquaducts) at the city of Zarach (Zārj, Iran), going back to 1000 BC, are still in use today (Anonymous http://enlightenme.com/ancient_plumbing). Later the Greek of Athens and Asia Minor and the Romans developed an indoor plumbing system for water supply, raising suspicion though about the large-scale lead poisoning in Roman culture. Around the same period in China, the Qin and Han Dynasties (200 BC–200 AD) were familiar with the use of plumbing, using bamboo instead of lead pipes. The Mayans in Central-America, and not the Spanish conquerors, also used an indoor system of pressurized water to construct the first known flush toilets (at the Maya city of Palenque, Mexico) (French and Duffy 2010). However, for many centuries much of these technologies - being lost during the Middle Ages - had disappeared from large regions of the world, including from North-Africa, Europe and Central-Asia (Anonymous http://enlightenme.com/ancient_plumbing). Therefore, it is reasonable to assume that the cumulative effects of increased hygiene measures would only appear in the last few centuries, especially in the West. Because although piped water may have been around for centuries, its access for the mass of people was achieved only recently.

Clean sanitation and safe drinking-water aren’t the only characteristics of the modern house, although sustainable access to safe drinking-water is an important one, according to the Unesco and the United Nations (UN) Millennium Development Goals and Targets (UNESCO http://www.unesco.org). Since the domestic space is also the environment where the children are raised during their first years till adulthood (or life-long), it is the domestic environment that has the primary influence on the shaping of the immune system. Chang classified the antigen exposure of young children into five patterns: ‘primitive’, ‘pre-modern’, ‘early modern’, ‘modern’, and ‘ultra-modern’ (Chang 2014). The ‘primitive’ pattern refers to conditions where children are staying in open shelters and are in absence of human waste disposal and sewerage systems, and also lacking soap, detergents, disinfectants, antibiotics and vaccines. These conditions today may only exist in remote, isolated sites in underdeveloped countries. In these conditions, helminth infections are abundant and allergic diseases virtually absent (Chang 2014). Generally, most children in underdeveloped countries and in poorly developed regions in developing countries have a ‘pre-modern’ pattern of antigen exposure (Chang 2014). In the ‘pre-modern’ pattern, access to a number of hygiene measures is limited, but still a lot of helminthic infections occur. From the ‘early modern’ pattern onwards, the helminth infections have disappeared (in Europe and the USA (Bleakley 2007) the deworming programmes were introduced in the first half of the twentieth century). On the other hand, however, the likelihood and severity of allergic diseases has strongly increased with ongoing modernization. Even until in recent decades, in countries like China, India and most of the developing countries, the lack of separated facilities for sewage and drinking water was very common. Nowadays, the use of chemically treated, sterile tap water in the ‘modern’ pattern and the lack of contact with natural water and soil in the ‘ultra-modern’ environment, are accompanied by a further increase in prevalence and severity of allergic diseases (Chang 2014). This is the situation which is most likely to be found among the richest households in the most ‘developed’ regions of developed countries. The finding that also domestic animals may develop symptoms of allergic diseases corroborates the viewpoint that skewing antigen exposure is a likely candidate for the increase in allergy prevalence, and obviously not the changes in child and adolescent behaviour as suggested by Platts-Mills (2015) (like more time spent on television watching and computer gaming) (Platts-Mills 2015).

#### Antigen Exposure and Living in (Ultra)Modern Environments

Whereas contacts with microbial species are virtually absent in these (ultra)modern environments, a few environmental antigens are persistently sticking to the presence of humans: (a) the common dust mite (*Dermatophagoides pteronyssinus* and *D. farinae,* from Europe and America respectively), that is living on skin cells people have shed; and (b) the common pets, like dogs and cats, shedding dander (dead skin from dogs, cats and other warm-blooded animals). In allergic reactions to dust mite, it is often not the mite itself but proteins in their droppings which cause the allergy. Dust mites prefer a warm and humid environment, which is rich in human skin remains and are mostly found in bedding, upholstered furniture and carpeting. Because people tend to breathe more deeply during sleep, allergies to dust mite antigen and also to pet dander are the most frequent causes of asthma.

On the other hand, milder hypersensitivity ailments of the respiratory system, like allergic rhinitis, are often caused by antigens caught during outdoor activities, when the breathing is swifter and less deep. These outdoor antigens in a modern, urban or cultivated environment often belong to also a limited number of species (see Sect. 3.4 below). Very well known are the pollen from birch (*Betula* sp.) or from Japanese Hemlock or Tsuga (*Tsuga sieboldii*) trees, or the grass pollen (Timothy grass, *Phleum pratense*, or Bermuda grass or Dog’s Tooth grass, *Cynodon* sp.). Also here, the hypersensitivity is not directed towards the pollen as a whole, but to specific proteins present in or released from the pollen at a certain time.

#### Antigen Exposure and Living in Rural Communities

Few studies have addressed the prevalence of allergic diseases in ‘primitive’ and ‘pre-modern’ communities, precisely because here the prevalence is very low or absent (Chang 2014). However, a striking connection between a low prevalence of autoimmune diseases and the living conditions in a rural community in Nigeria was already reported in Greenwood (1968). Moreover, high levels of serum IgE were found in rural areas in Venezuela (Lynch et al. 1998) and Indonesia (Hamid et al. 2013), corresponding with low prevalences of allergic diseases too (Chang 2014). On the contrary, the incidence of these disorders in the United States of America and Europe appears to have doubled in some decades of last century, especially during the 1960s and 1970s (Rook and Brunet 2005). The asthma prevalence rate among Taiwanese children was only 1% when the first recordings were made in the 1980 s, but nowadays its rate has raised to about 15% (Chang et al. 2007). The prevalence of asthma in children of the USA has further increased by 38% between 1980 and 2003 (Versini et al. 2015).

**Fig. 1:**
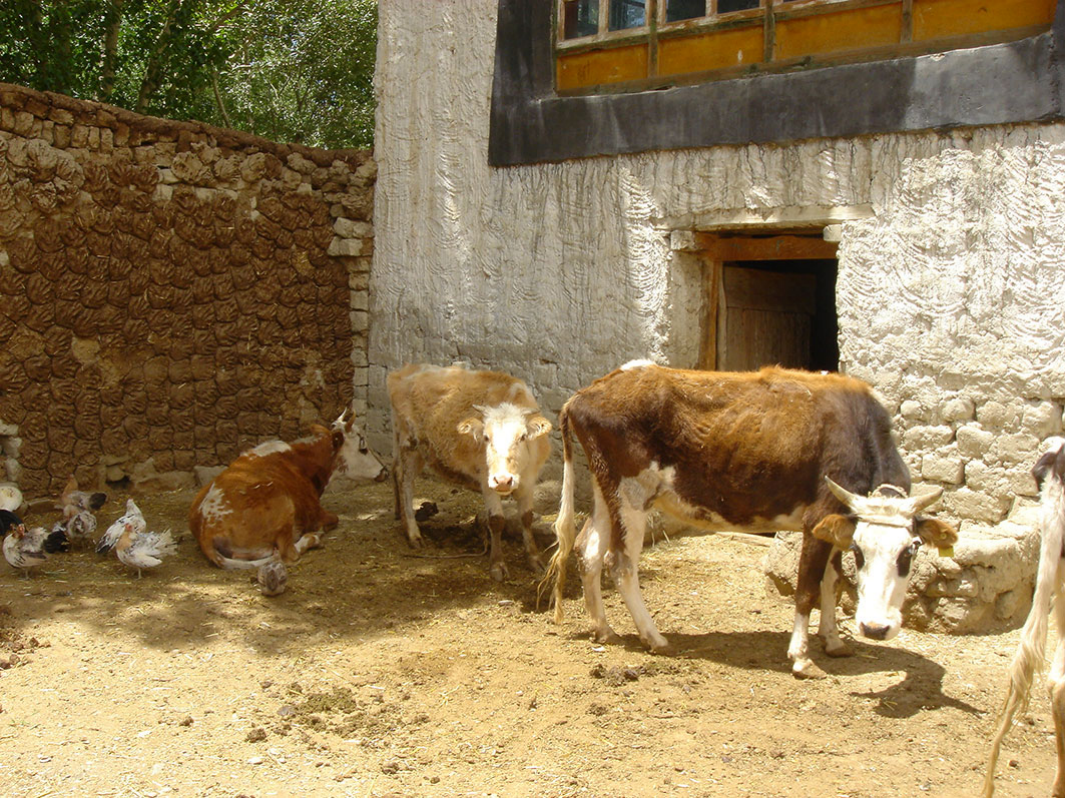
Photograph of antigen-rich environment in a combined farm and living shelter in Tibet Autonomous Region. Cattle dung is dried and kept as a fuel for house warming and for insulation. Horse, cattle and other domestic animals live in the ground floor and people on the second floor. Allergic diseases are absent in this quasi-underdeveloped region (Photo by Tse Wen Chang)

**Fig. 2:**
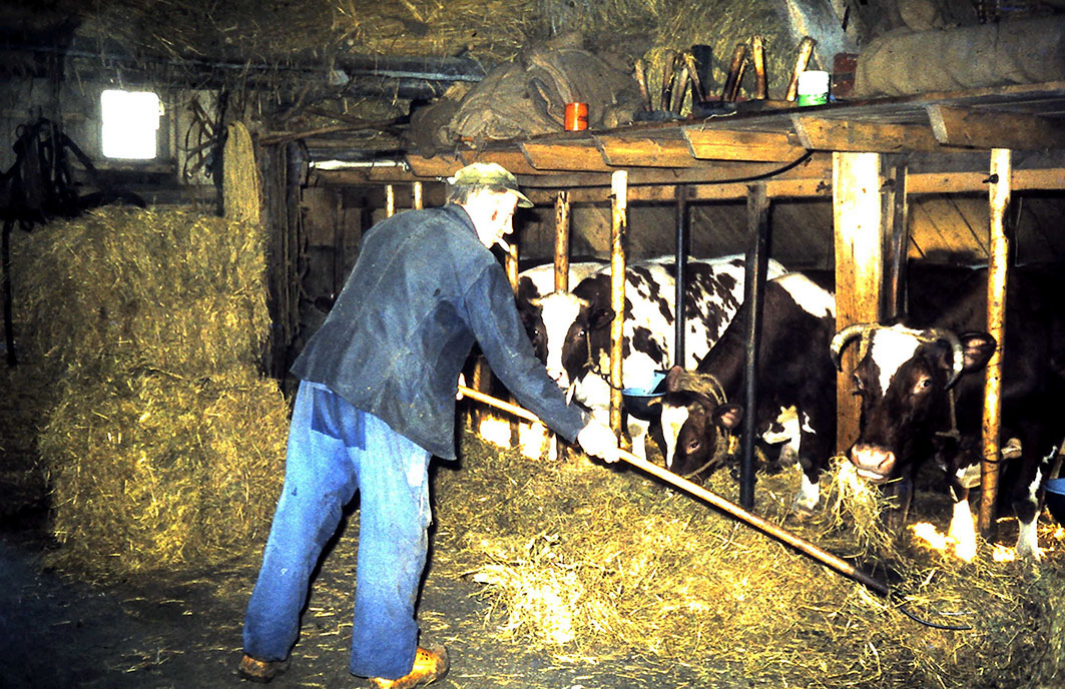
In recent history in a western country (e.g. in the Netherlands) too the farmers on the countryside lived in close proximity to their cattle, both living in the same building (Photo by Johan Alferink, Historical working-group, 1976)

Until recently, both in developed and developing countries, a variety of interspecies contacts would occur within the domestic environment (Fig. 1). In the urban environment, especially the older districts of town, old houses would harbor a variety of vermin animals (rats, house mouse, cockroaches, flies, fleas and lice), but due to the moisture and lack of ventilation, also many fungi and molds. On the countryside, moreover, especially in colder climate zones, in old farm homes a part was even reserved to shelter the cattle and poultry (Fig. 2). So the farmer could easily milk the cows and collect the eggs without leaving his farm. This form of cohabitation is often still the case in underdeveloped regions. With the cattle and the poultry living indoor, a lot of parasites would become in close contact with humans, in addition to a greater abundance of vermin animals. Moreover, living in tightly enclosed shelters enhances the exposure to indoor antigens (Chang 2014).

### Changes in Food Habits

The contacts with microorganisms and foreign antigens not only occur via the skin and respiratory tract, also the digestive tract plays a prime role as a delineation line between the inner circulation and the intestinal ‘outer’ world (see also Sect. 2). The lining enterocytes of the small intestine are not only in contact with food and IgA molecules, but they are also very close to the lymph nodes of the immune system (e.g. Peyer’s patches). Since the first humans succeeded in controlling fire, the control of food processing has been an important aspect of human culture, of public health, and hence also of the human immune system.

#### Changes in Food Processing

Several methods have been used from archaeological times till recent history. Drying fruits and vegetables was already known around 12,000 years ago, which method in later cultures was also applied to fish, game and meat from domestic animals (Nummer 2002). In medieval times until recently, also curing with salt (sodium chloride, but also nitrates), sugaring, pickling and smoking of foods were common practices. From the nineteenth century on, and especially in the twentieth century, after the venue of electric refrigerators and freezers, cooling, freezing, but also canning, heating (according to the temperature path followed called pasteurizing, after Louis Pasteur [1822–1895], or sterilizing of [canned] foods) became standard techniques. Since the second industrial revolution in the twentieth century, also irradiation, vacuum packing and the use of artificial food additives are used widespread in the industrialized world.

All these methods are based on the concept of killing the microorganisms or avoiding contact between microbes and the food products. In contrast to the former methods, in food fermentation (using yeasts) a very different strategy has been followed. Fermentation has also been an important alternative to combat food degradation in several ancient until modern cultures, and became very popular in particular for the production of alcoholic drinks (beers, wines…). This was mainly because the alcohol would kill the unwanted microorganisms. Fermentation, however, appeared to be essential for the production of dairy products like cheese too, and in some regions was and is still used in many more applications (like the use of fermented beans, soybeans and cabbage in contemporary China and Taiwan). Historical sources and the absence of a word for cabbage in Sanskrit and other ancient Eastern languages suggests the relatively young history of these typically winter foods in Central- and East-Asia. Probably, these winter foods were introduced following the trade with European merchants. Fermented cabbage, also known as the German term ‘Sauerkraut’ is well known both in Central and Eastern Europe as well as in Northern China (‘suan tsai’). Fermented tofu (from soybean cream) is a conspicuously odorous ingredient very popular in local Chinese cuisine.

The most dramatic change in food processing, however, may result from rapid change in the food preferences and taste of the young generations. It is well known that children are easily tempted to follow commercially advertized new food products, both in the East as in the West. The government control on advertisement campaigns (e.g. for highly sugared snack foods or for high fructose syrup) has often been proven to be rather ineffective and slowly running behind the facts (Aksoy and Beghin 2005). As a result, the changes in food habits tend towards increasing the preferences for so-called ‘fast food’ products with a very limited exposure to environmental antigens.

#### Changes in Food Traffic and Regulations

Following the increased globalization of trafficking of people and food products, an intensification of intercontinental regulations regarding the use of fermented food products has been noticed. For instance the American Food and Drug Administration (FDA) (Scott-Thomas 2010), the European Food Safety Authority (EFSA) (EFSA 2009) and the UN organizations for Food and Agriculture (FAO) have all provided extensive guidance measures and regulations. In many cases these regulations are directed against specific groups of molecules or pathogenic organisms possibly occurring in some fermented foods. For instance, they aimed to constrain the spread of microorganisms and to destroy in particular pathogenic species like *Clostridium botulinum*, *Escherichia coli*, *Salmonella* sp., *Listeria* sp., etc. (Scott-Thomas 2010). Targeted molecules are e.g. the group of biogenic amines (histamine, tyramine, 2-phenylethylamine but also the aliphatic, degradative products like putrescine and cadaverine, e.g. in wine and cheese fermentation, resp.) (EFSA 2011). Although these molecules may not be toxic *in se*, at high doses they may constitute a health risk when their metabolism is hampered. These bioamines are either metabolized by oxidative deamination by diamino oxidase (DAO) or by ring-methylation by histamine-N-methyltransferase (HNMT) (the HNMT pathway also involves the monoamine oxidase, MAO). Bioamine metabolism could be hampered in patients suffering from DAO deficiencies (Maintz and Novak 2007), or could be due to alcohol-induced reduction of DAO activity or to drugs containing DAO-inhibitors (Sessa et al. 1984). As a result histamine intoxication may follow, sometimes resulting in allergic symptoms and even asthma (Sattler et al. 1988).

#### Putative Beneficial Effects of Fermented Foods

Although the use of fermented foods is a much debated topic (see above), there is a growing consensus on the beneficial effects of certain groups of fermented diary products, also called pro-biotics. Moreover, contrary to the adversary effects of lactose-containing milk products, at least for lactose-intolerant subjects the overall effect of (fermented) milk products (with or without lactose) is considered to be positive: for instance, they may alleviate certain inflammatory reactions (Hosoya et al. 2012), whereas deprivation of fermented foods is associated with a fall in innate immune responses (Legrand and Mazurier 2010) and adverse immunological effects (Marcos et al. 2004; Ebringer et al. 2008; Tsai et al. 2012). Many studies reported that lactic acid bacteria as *Lactobacillus* and *Bifidobacterium* are effective at enhancing innate and adaptive immunity (Legrand and Mazurier 2010; Tsai et al. 2012), although the mechanisms appear to involve a wide variety of molecules and pathways: from lactoferrin, released from neutrophils upon antigen-nonspecific stimulation (Legrand and Mazurier 2010), to the secretion of polymeric IgA (Tsai et al. 2012), and of IL-17 (Hosoya et al. 2012), milk oligosaccharides and other small dairy molecules (Ebringer et al. 2008) and, finally, the modulation of Dendritic Cell/Natural Killer (DC/NK) cell interactions and a balanced T-helper cell response (Tsai et al. 2012). Also here, changing food habits may either improve or impoverish the beneficial effects of fermented dairy products. The latter may result from the impact of heat processing on the biological activity of milk products (reviewed in Ebringer et al. 2008). In conclusion, it is due to a balanced intestinal microflora and a diversity of biogenic protective molecules that the host’s immune system may function in optimal conditions; the reduction of diversity in microbial species and molecules generally reduces the immune defense capacities of the host.

In contrast to the extensive literature on the immune effects of fermented milk products, few studies have addressed the immune effects of fermented cabbage or fermented vegetables altogether. Interestingly, in animal studies a beneficiary effect of cabbage fermentation extract was found on the immune system of Sprague-Dawley rats (Miyazaki et al. 2001).

### The Changes in Biodiversity and Outdoor Environment

Although much debated, the changes in biodiversity and changes in the biological determinants of the outdoor environment are much harder to establish than the indoor and food ‘environmental’ determinants. The main reason is that it is still very difficult to establish the biodiversity in terms of the total number of biological species – and especially the abundance of this innumerable amount of species - that surround us. Numerous new marine species are discovered every year, as counted by a worldwide consortium, called the *Census of Marine Life*, organized since 2000 (Assubel et al. 2010). For the microbial organisms the biodiversity may even appear far greater and incredibly harder to establish. For in the case of microorganisms, including protists, bacteria and archaeobacteria, one must rather speak about phylotypes than about different species, because of the huge genetic diversity and exchangeability of genetic material.

A similar situation holds for the soil biology and microbiology (de Neergaard 2005). Although soil organisms constitute only a tiny fraction of the total soil, their diversity is enormous compared to other habitats on earth, and the characterization of the populations and their dynamics is still in its infancy (de Neergaard 2005). Microbiota form the dominating group, consisting of archaea, bacteria, actinomycetes, fungi, algae and protozoa. However, Chang and Pan (2008) have extensively reviewed the impact of modern civilization upon the contact between humans and soil organisms. Especially in young children, the habit of ‘eating soil’ or geophagy (by direct or indirect contact between soil and mouth), has been largely reduced in postmodern culture, due to an increased cleanliness, the lack of direct soil contact in the domestic environment, and following the efforts of the education process.

There are of course many serious concerns about the decline in biodiversity in both marine and terrestrial habitats on earth. These biodiversity concerns mostly regard the macrobiota and especially the much endangered classes of large mammals, birds, fish and in particular also many amphibians and reptiles (Council of Europe 1979; United Nations 1992). In particular, the UN concern about biological diversity is that it is significantly reduced by ‘certain human activities’, and the UN notes that it is ‘vital to anticipate, prevent and attack the causes of significant reduction or loss of biological diversity’ at its source (United Nations 1992).

From the immunological point of view, these concerns about a presumed declining biodiversity in the human environment are particularly of interest because of their symptomatic value. Since it is the total of human activities that cause a significant loss of biological diversity (United Nations 1992), biodiversity therefore is most likely to follow a decline due to human activities and especially in human-controlled habitats. The areas that are most controlled by human activities, are the urban areas and intensively used agricultural environments (Duro et al. 2014). So far, there have been few and too limited successes with regard to the European attempts to preserve the biodiversity of the natural environment (EIONET 2015). Moreover, there is also a lot of criticism on the reinforcement of the strategical *Natura 2000* programme, and the ongoing concern is that these measures are more directed towards the creation of circumscribed and limited areas with the status of a natural reserve, and are not aiming at the preservation of the ecological stability and biodiversity of our everyday environment. Practically, there is little or only fragmentary information available on how human activities, and especially human agriculture in an economically and industrially globalized environment, is rapidly changing the immediate outdoor surroundings of humans (Duro et al. 2014; McKinney 2008; Riley et al. 2005).

To conclude this paragraph, the enormous changes in microbial diversity of the environment, since the control of fire by the early humans, are most significantly observed in the domestic environment. Serious concerns, however, are substantially documented based on the changes in outdoor environment and food consumption. For the adaptive immune system, and especially for the development of allergic reactions, the domestic and immediate, local environments are most important.

## Role of Helminthic Parasites and ‘Skewed Antigen Exposure’ Hypothesis

Although the exact mechanism according to which helminthic parasites stimulate the immune response is yet unknown, it is a well-established fact that in populations with high incidence of helminthic infection the serum IgE is much enhanced (see Sect. 2). According to Rook and others (Rook and Brunet 2005; Versini et al. 2015), the presence or absence of ‘old friends’ also involves other immune responses, like inflammatory bowl disease (IBD). According to Rook and Brunet (2005), the ‘old friends’ is a group name for commensal species, especially helminths and also other ‘relatively harmless’ microorganisms like saprophytic mycobacteria and lactobacilli. So, also autoimmune disorders should be included in the Hygiene and ‘Old Friends’ Hypotheses (Rook and Brunet 2005; Versini et al. 2015). A classic hypothesis is that helminths (or helminth products) may promote a T-helper 2 (Th2)-type response and down-regulate Th1/Th17 differentiation, leading to increased Th2-cytokine (IL-4, IL-5, IL-9, IL-10, IL-13) and decreased Th1-Th17-type cytokine (TNF-α, IFN-γ, IL-6, IL-12, IL-17) secretion (Versini et al. 2015). Both groups of cytokines are also labeled as ‘tolerogenic’ versus ‘inflammatory’ mediators. Th17 cells are a recently-defined subset of T-helper cells primarily secreting IL-17, that would exert a key-role in developing autoimmune disease (Versini et al. 2015; Bettelli et al. 2007). However, according to Rook and Brunet (2005), since an individual may develop a Th1 or Th2 mediated inflammatory disorder, in the absence of optimal levels of immunoregulation and depending on his own Th1/Th2 bias and immunological history, the Th1/Th2 balance is not instructive (Rook and Brunet 2005; Versini et al. 2015).

Therefore, a ‘skewed antigen exposure’ hypothesis was proposed by Chang (Chang and Pan 2008; Chang 2014), following the principle of finding the most parsimonious explanation to interprete the clinically observed data. In order to obtain sufficient cross-linking of bound IgE’s and their high-affinity receptor (FcεRI), the density of IgE/ FcεRI specific for the sensitizing antigen must be above a certain threshold (MacGlashan et al. 1997). The proportion of FcεRI that is occupied by IgE of a particular, ‘episode-relevant’ antigen, moreover, is related to the proportion of that IgE to total IgE in the blood circulation under steady-state conditions (Malveaux et al. 1978). Therefore, lowering the total IgE in serum, as a result of the absence of helminths or so-called ‘old friends’, may become sufficient to explain an increased ratio of IgE’s specific to innocuous environmental antigens to total IgE (Chang and Pan 2008; Chang 2014).

For instance, in some children with food allergies the serum IgE specific for the allergens may account to more than 10% or even 50% of total serum IgE (Schneider et al. 2013). When looking at the proportion of ‘episode-relevant’ antigen-specific IgE to total IgE, it is therefore conceived that this proportion is critical for the development of an allergic reaction (Chang 2014). Accordingly, it has been analyzed and reported that the patients that are the most difficult to treat with the IgE-neutralizing antibody drug omalizumab [Xolair ^®^, directed against the FcεRI-binding site (Chang 2000; Chang et al. 1990)], are those where antigen-specific IgE amounts to 3–4% of total IgE (Johansson et al. 2009). Given that a proportionality exists between the proportion of antigens to which an individual is exposed and the proportion of IgE’s specific to these antigens in the individual’s serum, this would correspond with an availability of less than 25–30 (in absolute numbers) dominant but innocuous antigens in a ‘clean’ environment (i.e. with a low contact level with environmental biodiversity). The more the indoor environment is tightly shut off from the outdoor environment, the fewer the number and diversity of outdoor antigens to which the children are exposed (Chang 2014).

## Summary: The Hygiene Hypothesis, the Old Friends Hypothesis, and the Skewed Antigen Exposure Hypothesis Are Required to More Fully Explain the Rise of Allergy in Modern Times

The Hygiene Hypothesis emphasized the decreased microbial infections in early childhood and their impacts on the regulatory T cells. The Old Friends Hypothesis stresses the abolition of the commensal helminthes from our bodies and their impacts on various regulatory aspects of the immune system. The Skewed Antigen Exposure Hypothesis stresses the drastic changes of the environmental antigens in our living shelters and their impacts on IgE, mast cell activities, and other immune activities. This article elucidates that the Hygiene Hypothesis, the Old Friends Hypothesis, and the Skewed Antigen Exposure Hypothesis not only are coherent, but they are also required to fully explain the rise of allergy in modern times.

There is ample evidence that the frequent microbial infections tune up the Th-1 response and tune down Th-2 response, including IgE-mediated pathway, which manifest allergic symptoms. As a proof of the involvement of helminths in balancing the immune system, several studies, including organized clinical trials, have shown that re-infections with various species of helminthes can alleviate symptoms of severe asthma and inflammatory bowl disease (Versini et al. 2015). The methods used in those studies varied from colonization by helminthes larvae and oral administration of worm ova. Furthermore, it is a reasonable speculation that if the pattern of environmental antigens in our living environment were not as skewed as it is, the allergic reactions would probably not occur.

The detailed analysis on the impacts or respective influences on the immune system of the three hypotheses as presented in this article, is meaningful. Such an analysis may help devise approaches to deal with the increasingly serious allergic problems. An interesting hypothetical scenario is that if we were able to return to the living environment and lifestyle of people living in an advanced city, such as London, in the beginning of the nineteenth century, we would not have allergic problems. However, a return to the frequent microbial infections and helminth infestation would be too much to bear. It seems that the only acceptable remedy would be to become exposed to the variety and amounts of environmental antigens as people did 200 years ago.

An intriguing thought is that, assuming the present Skewed Antigen Exposure Hypothesis is correct, whether the immune imbalance in people affected by allergy or people at-risk in developing allergy (caused by the skewed exposure to environmental antigens) can be amended by somehow exposing those individuals to large amounts of harmless environmental antigens. The specific questions will be how such an exposure to large amounts of harmless environmental antigens could be designed and administered, and how the anticipated immune effects could be tested in animal models and eventually in human subjects.
